# Supplementary material for: Evolutionary repair reveals an unexpected role of the tRNA modification m1G37 in aminoacylation
Source: Nucleic Acids Res. 2021 Nov 11;49(21):12467–85. doi: 10.1093/nar/gkab1067 (PMC8643618; doi:10.1093/nar/gkab1067)
Supplement: gkab1067_Supplemental_Files [file gkab1067_supplemental_files.zip › Supplementary Information - NAR - revision.pdf]

Supplementary Information

Evolutionary repair reveals an unexpected role of the tRNA modification m^{1}G37 in aminoacylation

Ben E. Clifton, Muhammad A. Fariz, Gen-Ichiro Uechi and Paola Laurino

Supplementary Figure 1. Design of luciferase reporter constructs. The linker sequence was designed to include a TAA stop codon in the +1 frame, a frameshift reporter sequence (CCC-C, CCC-T, or CCC) in the 0 frame, a TAA stop codon in the 0 frame, and a Gly-Ser-Ser linker in the +1 frame, in that order. The remaining nucleotides were chosen to optimize codon usage frequency (i.e., to avoid rare codons) and amino acid solubility. The vector map was generated in SnapGene software.

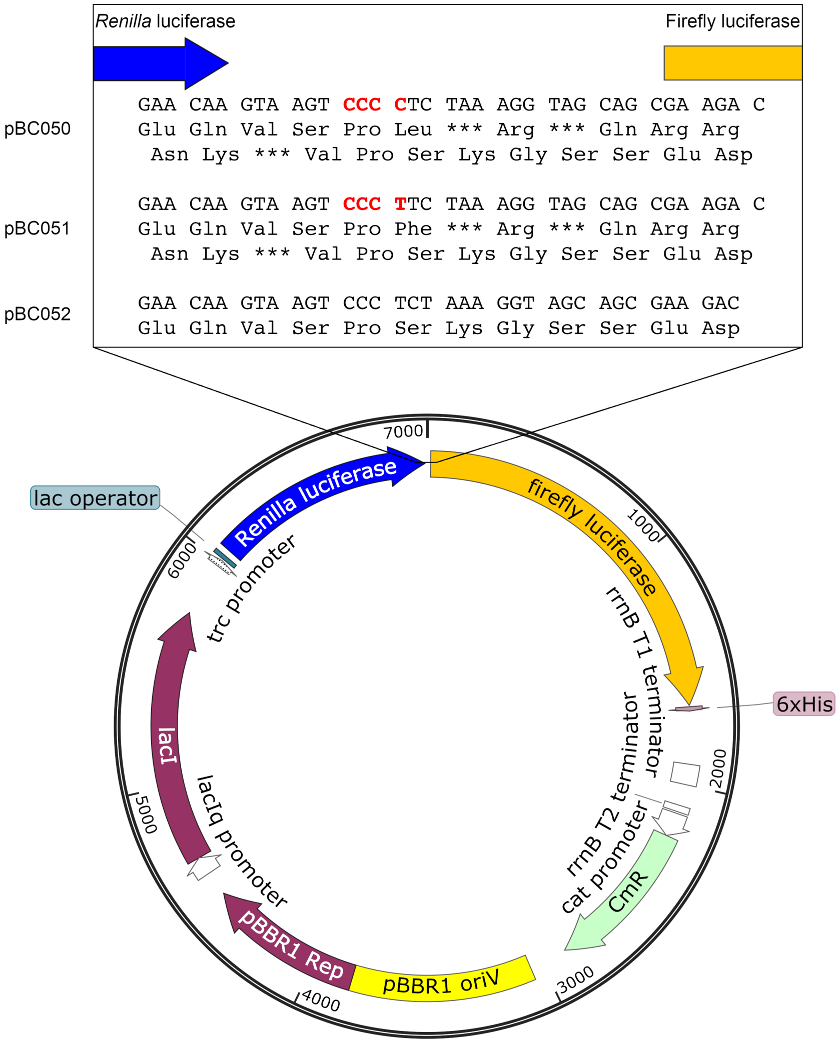

Supplementary Figure 2. Barcode mutations do not affect growth of *E. coli* BW25113. Growth assays of the WT BW25113 strain (black) and the seven barcoded strains (gray; see Supplementary Table 1) in LB media at 37 °C. Data represent mean ± s.d. of three biological replicates (different colonies).

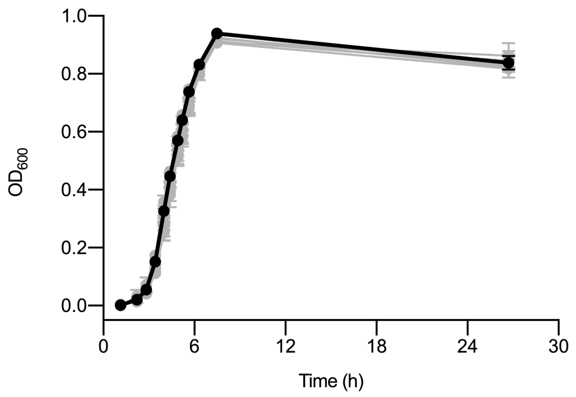

Supplementary Figure 3. Growth rates of key *E. coli* strains and evolved populations. For each strain or population, the mean and individual replicate values are shown, except in panel **H** where mean ± s.d. is shown (*n* = 12 to 24). The dotted lines indicate the growth rate of the WT strain (1.53 h^{-1}) and the mean growth rate of the R154A, S165L, D169A, and Y86* strains (0.54 h^{-1}). The precise number of replicates for each strain is indicated in Figures 1-3. **A,** Strains with mutations in *trmD* (Fig. 1B). **B-F**, Evolving populations (Fig. 2). **B**, G117N; **C**, S165L; **D**, D169A; **E**, R154A; **F**, Y86*. **G**, Mutants of the *trmD* G117N strain with a second mutation in *trmD* (Fig. 3B). **H**, Mutants of the *trmD* R154A strain with a single mutation in *proS* (Fig. 3C and Supplementary Fig. 7). **I**, Mutants of the *trmD* R154A strain with mutations in *argV* and *argX* (Fig. 3D).

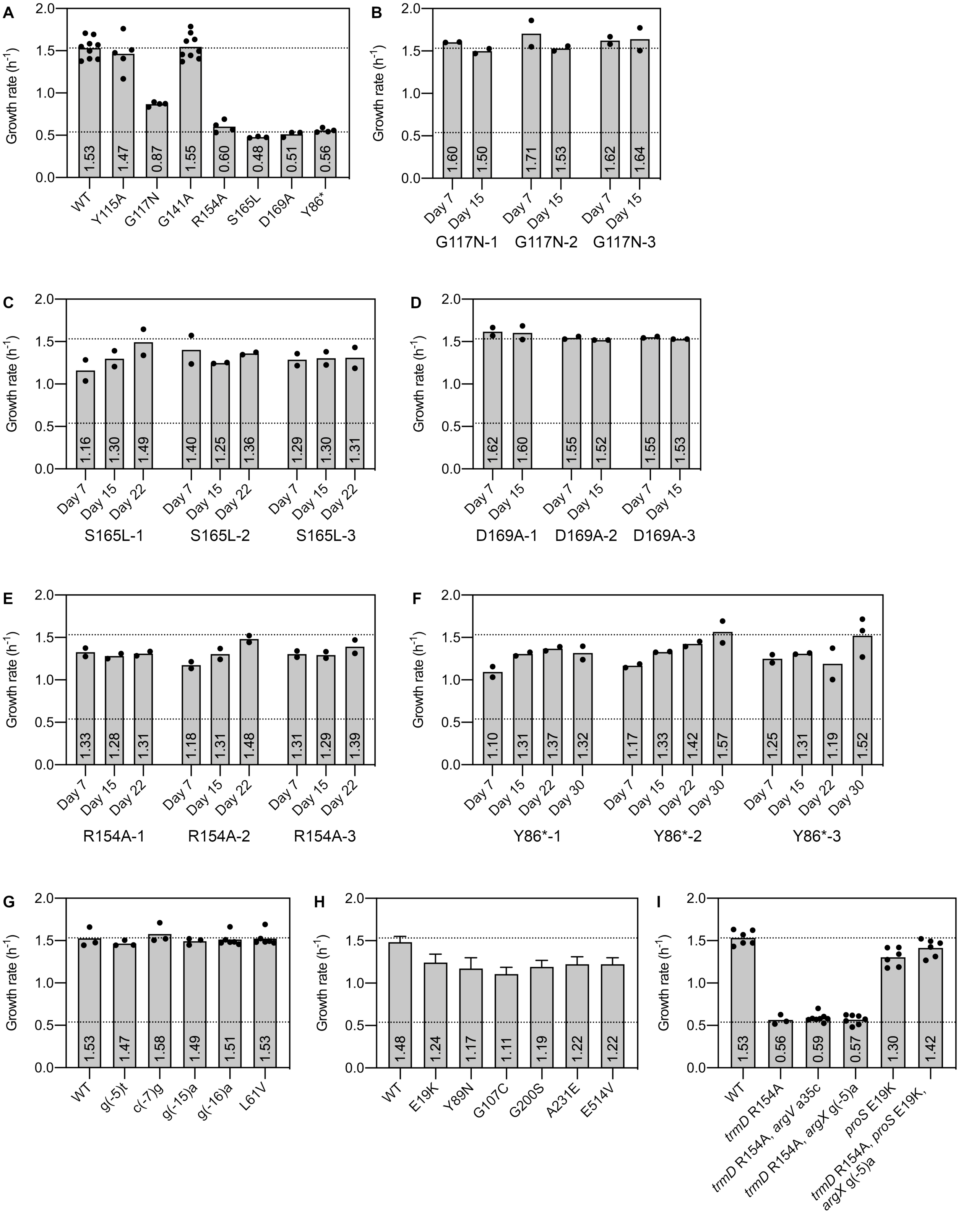
A
Growth rate (h^{-1})
2.0
1.5
1.0
0.5
0.0
1.53
1.47
0.87
1.55
0.60
0.48
0.51
0.56
WT
Y115A
G117N
G141A
R154A
S165L
D169A
Y86*
B
Growth rate (h^{-1})
1.60
1.50
1.71
1.53
1.62
1.64
Day 7
Day 15
G117N-1
G117N-2
G117N-3
C
Growth rate (h^{-1})
1.16
1.30
1.49
1.40
1.25
1.36
1.29
1.30
1.31
Day 7
Day 15
Day 22
S165L-1
S165L-2
S165L-3
D
Growth rate (h^{-1})
1.62
1.60
1.55
1.52
1.55
1.53
Day 7
Day 15
D169A-1
D169A-2
D169A-3
E
Growth rate (h^{-1})
1.33
1.28
1.31
1.18
1.31
1.48
1.31
1.29
1.39
Day 7
Day 15
Day 22
R154A-1
R154A-2
R154A-3
F
Growth rate (h^{-1})
1.10
1.31
1.37
1.32
1.17
1.33
1.42
1.57
1.25
1.31
1.19
1.52
Day 7
Day 15
Day 22
Day 30
Y86*-1
Y86*-2
Y86*-3
G
Growth rate (h^{-1})
1.53
1.47
1.58
1.49
1.51
1.53
WT
g(-5)t
c(-7)g
g(-15)a
g(-16)a
L61V
H
Growth rate (h^{-1})
1.48
1.24
1.17
1.11
1.19
1.22
1.22
WT
E19K
Y89N
G107C
G200S
A231E
E514V
I
Growth rate (h^{-1})
1.53
0.56
0.59
0.57
1.30
1.42
WT
trmD R154A
trmD R154A, argV a35c
trmD R154A, argX g(-5)a
proS E19K
trmD R154A, proS E19K, argX g(-5)a

Supplementary Figure 4. UPLC analysis of nucleoside content in *trmD* mutant strains. A, Separation of nucleoside standards. Left panel, UV chromatogram with detection at 260 nm; right panel, extracted ion chromatogram for m^1G ($[M+H]^+$ *m*/*z* 298). Abbreviations: A, adenosine; C, cytosine; G, guanosine; U, uridine; m^1G, N^1-methylguanosine; ψ, pseudouridine. **B**, Separation of digested $tRNA^{Pro}$ (CGG) purified from various *trmD* mutant strains of *E. coli*. Left panel, UV chromatogram with detection at 260 nm; right panel, extracted ion chromatogram for m^1G ($[M+H]^+$ *m*/*z* 298). The elution time of m^1G (16.198 min) is indicated by a dashed line. Data for the WT and Y86* strains are reproduced from Fig. 1D for comparison with the other strains. **C**-**E**, Evaluation of assay sensitivity. Digested WT $tRNA^{Pro}$ (CGG) was spiked into a mixture of 20 μM each of adenosine, cytosine, guanosine and uridine at the indicated concentration. **C,** A_{260} chromatogram of a 5% (v/v) dilution of digested tRNA. **D,** Extracted ion chromatogram of a 1% (v/v) dilution of digested tRNA. **E,** Extracted ion chromatogram for digested $tRNA^{Pro}$ (CGG) from the *trmD* Y86* strain, showing that m^1G is not detected with a limit of detection <1%.

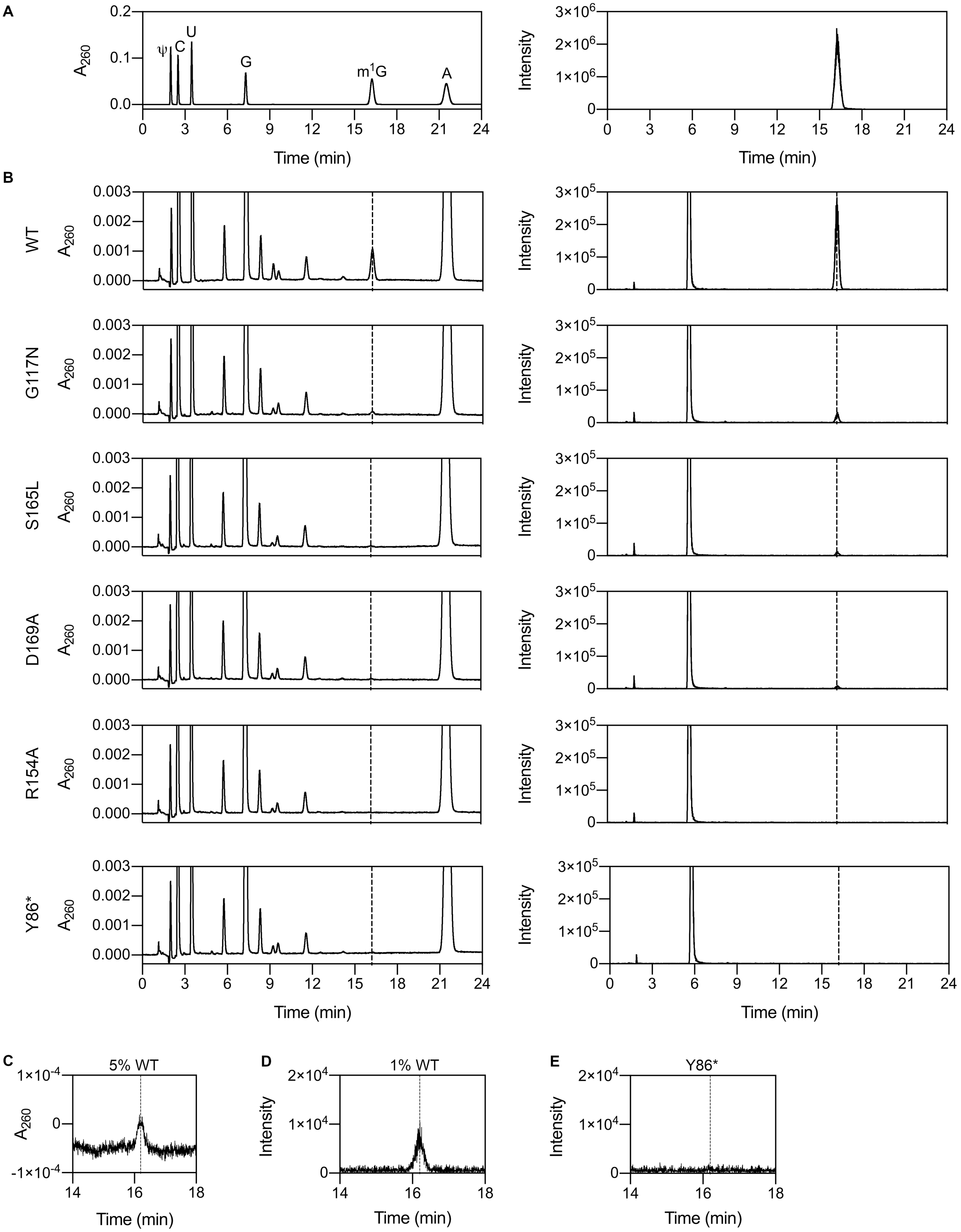

A
B
C
D
E
A260
Intensity
Time (min)
ψ
C
U
G
m1G
A
WT
G117N
S165L
D169A
R154A
Y86*
5% WT
1% WT
Y86*

Supplementary Figure 5. Copy number of *trmD* in evolving populations. Mean copy number of *trmD* in population genomic DNA extracted from the G117N, S165L, D169A and R154A populations at different time points was measured by qPCR. Error bars represent 95% confidence intervals. Estimates are derived from two technical replicates per primer pair for each sample.

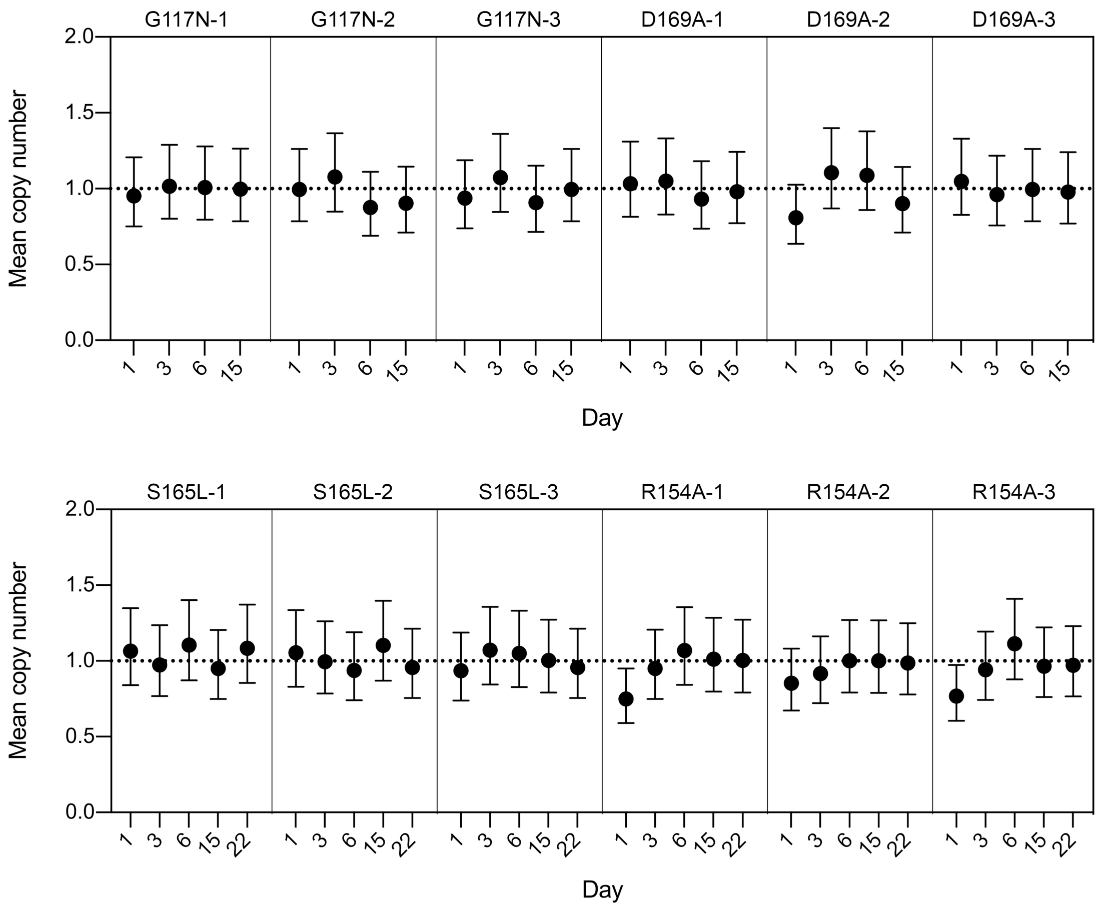

Supplementary Figure 6. Off-target mutations introduced during MAGE do not affect growth of *trmD* mutant strains. Growth assays of each strain transformed with the pBAD-yTrm5 and pFREE plasmids (i.e., the final strains to be stored as a glycerol stock before the evolutionary experiment) were performed in LB media containing 25 μg/mL kanamycin, 100 μg/mL ampicillin and 0.2% (w/v) glucose at 37 °C. Data represent mean ± s.d. of two biological replicates (different colonies).

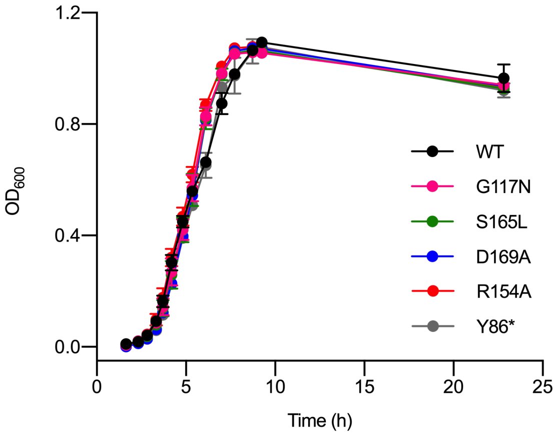

Supplementary Figure 7. Replicate growth assays of variants of the *trmD* R154A strain with an additional mutation in *proS*. Data represent mean ± s.d. WT, E514V, *n* = 3 colonies; G200S, A231E, E19K, Y89N, G107C, *n* = 6 colonies (three colonies each from two independently isolated strains). The E19K, A231E, and E514V variants show consistently faster growth than the Y89N, G107C, and G200S variants when the full growth curve is considered.

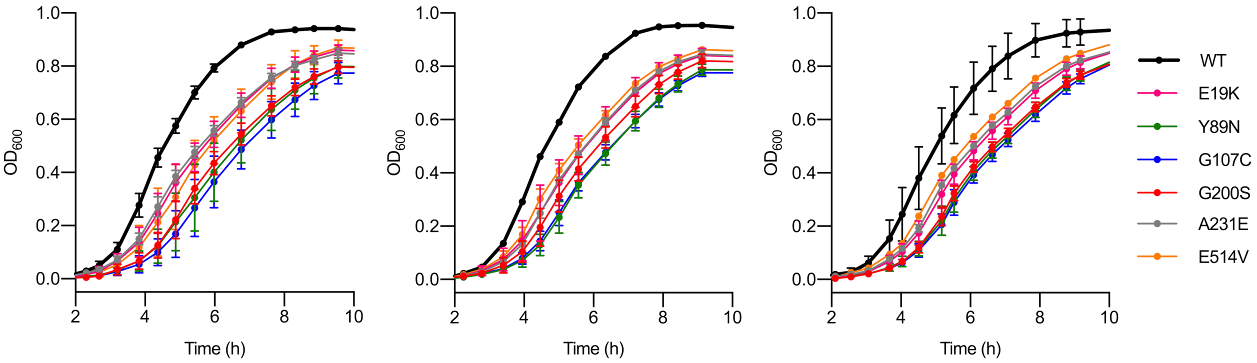

Supplementary Figure 8. Sanger sequencing analysis of *proS* at early time points in the evolutionary experiment. The *proS* gene was amplified from population genomic DNA at each time point for the S165L, R154A, and Y86* populations, and polymorphisms were identified by Sanger sequencing. Each sequencing trace shows three bases centered on the mutated base (not necessarily the mutated codon). Mutations are described relative to the antisense strand. The frequency of each mutation at day 9, determined by NGS, is also indicated.

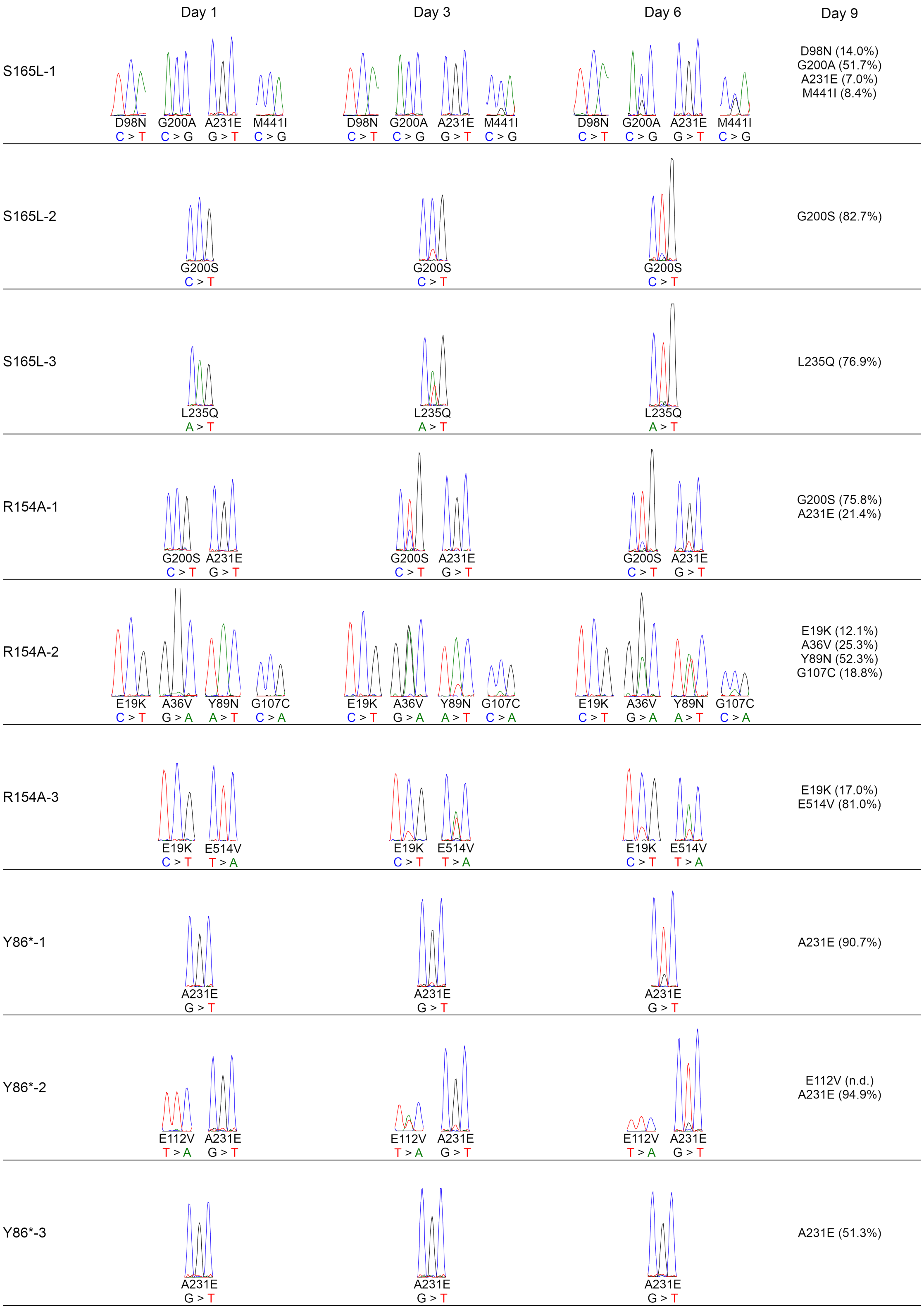
Day 1
Day 3
Day 6
Day 9
S165L-1
D98N C > T
G200A C > G
A231E G > T
M441I C > G
D98N (14.0%)
G200A (51.7%)
A231E (7.0%)
M441I (8.4%)
S165L-2
G200S C > T
G200S (82.7%)
S165L-3
L235Q A > T
L235Q (76.9%)
R154A-1
G200S C > T
A231E G > T
G200S (75.8%)
A231E (21.4%)
R154A-2
E19K C > T
A36V G > A
Y89N A > T
G107C C > A
E19K (12.1%)
A36V (25.3%)
Y89N (52.3%)
G107C (18.8%)
R154A-3
E19K C > T
E514V T > A
E19K (17.0%)
E514V (81.0%)
Y86*-1
A231E G > T
A231E (90.7%)
Y86*-2
E112V T > A
A231E G > T
E112V (n.d.)
A231E (94.9%)
Y86*-3
A231E G > T
A231E (51.3%)

Supplementary Figure 9. Duplication of *proS* observed in the Y86*-3 population at day 9. A, Boundaries of the duplicated region identified by new junction analysis. **B,** Validation of duplicated region by analysis of read coverage depth. The light gray region corresponds to the duplicated region, and the dark gray region corresponds to the *proS* open reading frame. The dotted line represents the mean read coverage depth across the whole genome.

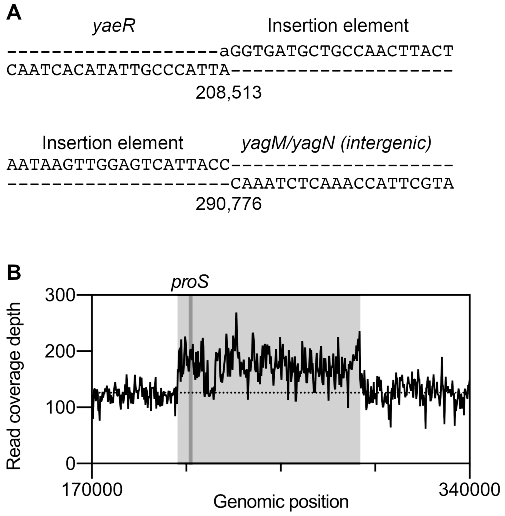

Supplementary Figure 10. Gel shift analysis of the aminoacylation activity of ProRS variants on m[1]G37-modified and G37-unmodified tRNA^{Pro} (CGG). Reaction conditions: tRNA concentration 2 μM, enzyme concentration 2.5 nM, reaction temperature 37 °C, reaction time 14 min. The enzyme concentration was chosen to ensure linearity of product formation for the WT enzyme with m[1]G37-modified tRNA over the course of the assay. + (1), positive control with 1 μM WT ProRS; + (2), positive control with 1 μM ProRS E514V; -, no enzyme control. The percentage of aminoacylated tRNA in each lane is indicated. Each panel (**A**-**C**) shows a single experimental replicate. The replicate shown in Fig. 5B corresponds to panel **C**. Raw gel images are provided in Supplementary Data 1.

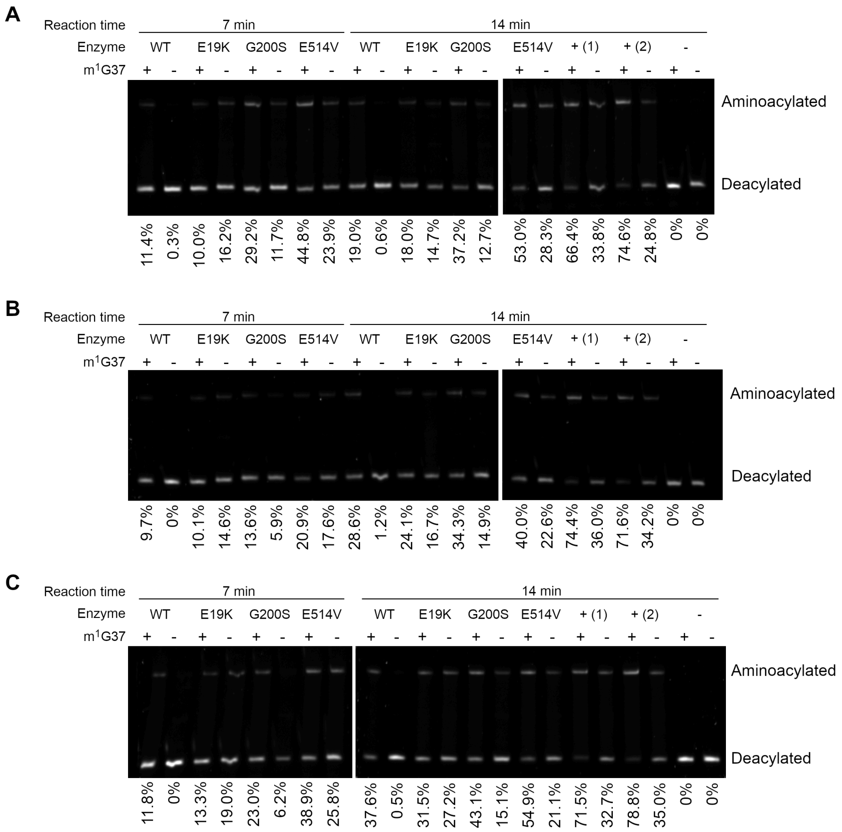

Supplementary Figure 11. Mutations in *proS* do not affect growth of *E. coli* BW25113. Growth assays of the WT BW25113 strain and variants with mutations in *proS* (E19K, G200S, or E514V) in LB media at 37 °C. Data represent mean ± s.d.; WT, *n* = 3 colonies; *proS* mutants, *n* = 6 colonies (three colonies each from two independently isolated strains).

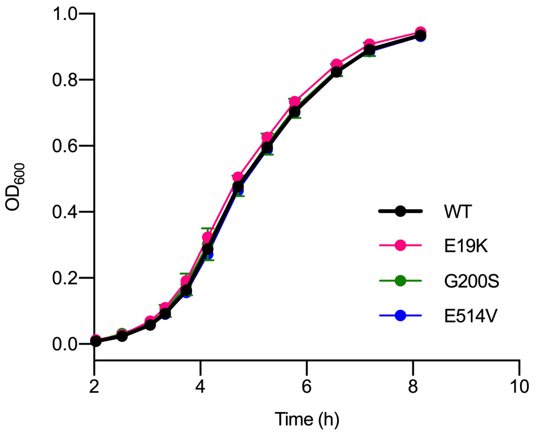

Supplementary Figure 12. Gel shift analysis for determination of Michaelis-Menten parameters for aminoacylation of m^{1}G37-modified and G37-unmodified tRNA^{Pro} (CGG) by WT and E514V ProRS. The rate of aminoacylation was measured using a 2-fold dilution series of tRNA. One representative experimental replicate (each with two technical replicates in the case of m^{1}G37-modified tRNA) is shown for each enzyme-tRNA combination. The percentage of aminoacylated tRNA in each lane is indicated. Raw gel images are provided in Supplementary Data 1. Abbreviations: n.q., not quantified; (+), positive control with 1 μM enzyme; (-), negative control with no enzyme. **A,** WT ProRS + m^{1}G37 tRNA. Enzyme concentration, 0.8 nM; maximum total tRNA concentration 6.29 μM. **B,** E514V ProRS + m^{1}G37 tRNA. Enzyme concentration, 0.267 nM; maximum total tRNA concentration 4.15 μM. **C,** WT and E514V ProRS + G37 tRNA. Enzyme concentration, 15 nM (WT) or 1 nM (E514V); maximum total tRNA concentration 3.70 μM.

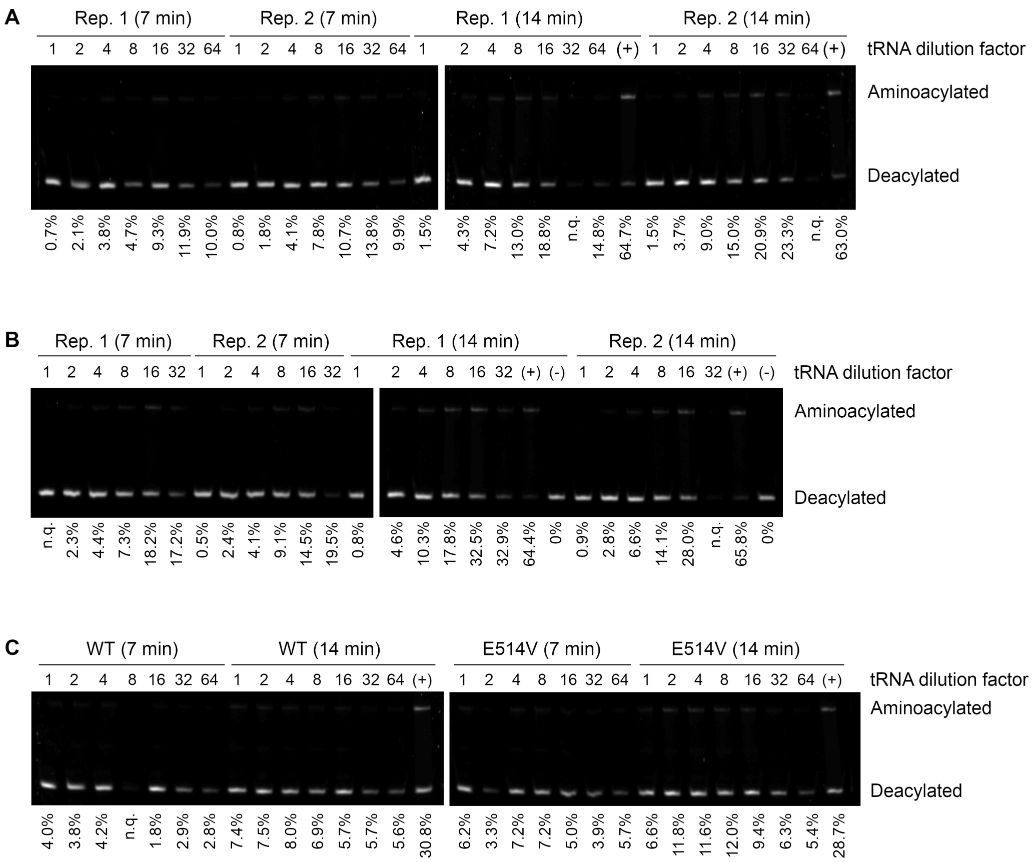

Supplementary Figure 13. Determination of +1 frameshift frequency in *trmD* mutant strains using a luciferase reporter assay. A, Ratio of firefly luminescence to *Renilla* luminescence in *E. coli* strains transformed with CCC-C and CCC-U frameshift reporter plasmids and a CCC control plasmid. Data represent mean ± s.d., n = 9 biological replicates, with individual replicates shown. Statistical significance was tested using a two-sided Student's t-test with a significance threshold of $P < 0.05$. CCC-C: WT vs. TrmD R154A, $P < 0.0001$; WT vs. TrmD R154A/ProS E514V, $P < 0.0001$; TrmD R154A vs. TrmD R154A/ProS E514V, $P = 0.2289$ (n.s.). CCC-U: WT vs. TrmD R154A, $P < 0.0001$; WT vs. TrmD R154A/ProS E514V, $P < 0.0001$; TrmD R154A vs. TrmD R154A/ProS E514V, $P = 0.1678$ (n.s.). **B,** +1 frameshift rates at CCC-C and CCC-U reporter sequences. Data represent mean ± s.d. (determined by error propagation), n = 9 biological replicates. Statistical significance was tested using a two-sided Student's t-test with a significance threshold of $P < 0.05$. CCC-C: WT vs. TrmD R154A, $P = 0.0015$; WT vs. TrmD R154A/ProS E514V, $P = 0.0003$; TrmD R154A vs. TrmD R154A/ProS E514V, $P = 0.1510$ (n.s.). CCC-U: WT vs. TrmD R154A, $P < 0.0001$; WT vs. TrmD R154A/ProS E514V, $P < 0.0001$; TrmD R154A vs. TrmD R154A/ProS E514V, $P = 0.1121$ (n.s.).

A

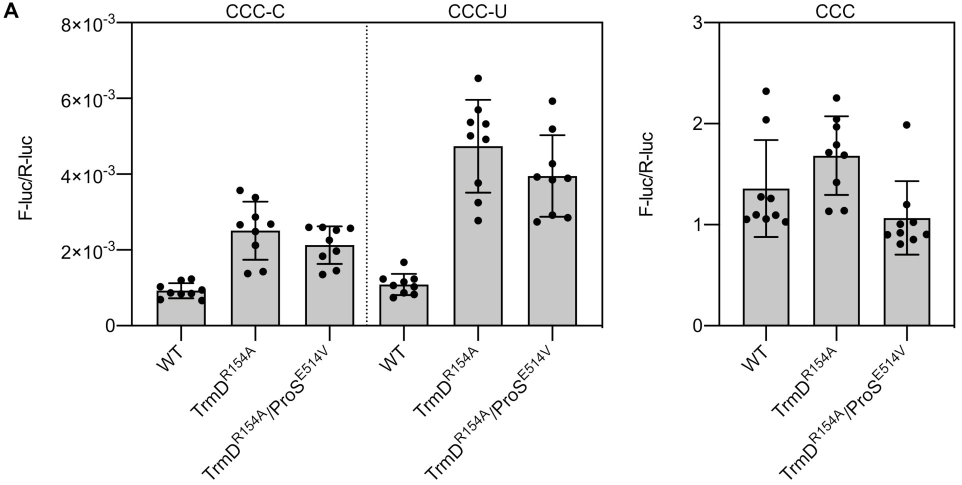

B

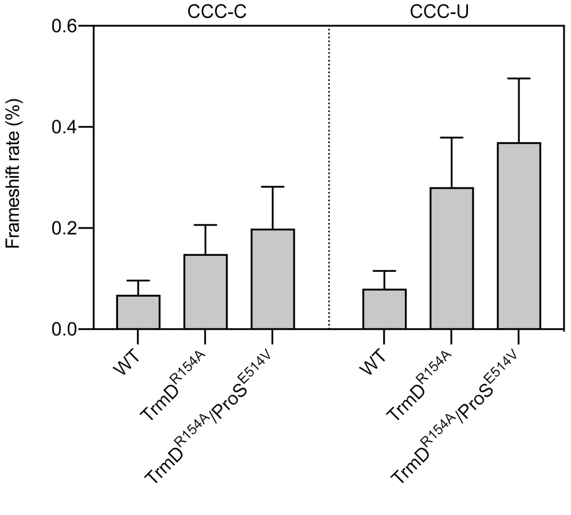

Supplementary Figure 14. Effect of mutations upstream of the *trmD* ORF on predicted mRNA secondary structure. A, Structure of *trmD* mRNA from the -30 position (immediately following *rimM*) to the +28 position, predicted using RNAfold version 2.4.18 (Lorenz et al., 2011). Positions where a mutation is observed in the evolutionary experiment are shown in light blue, and the start codon of *trmD* is shown in dark blue. The mRNA structure was visualized using Forna (Kerpedjiev et al., 2015). **B,** Effect of point mutations on predicted minimum free energy of *trmD* mRNA secondary structure (positions -30 to +28). Free energy calculations were performed using RNAfold version 2.4.18 (Lorenz et al., 2011).

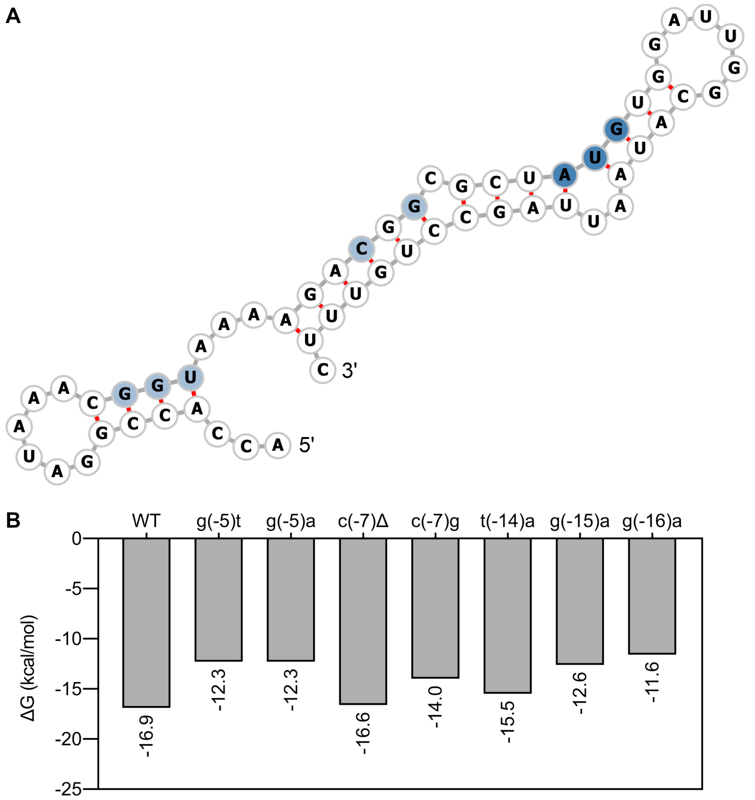

Supplementary Table 1. List of *E. coli* strains used in this study.

Strain	Genotype	Source	Comments
BW25113		National Institute of Genetics, Japan	
**Barcoded strains**			
BC_S049	BW25113; 842706_842710 delins AGCGC	MAGE	
BC_S050	BW25113; 842706_842710 delins ATAGC	MAGE	
BC_S052	BW25113; 842706_842710 delins AAGGC	MAGE	
BC_S054	BW25113; 842706_842710 delins ACGTC	MAGE	
BC_S055	BW25113; 842706_842710 delins AATAC	MAGE	
BC_S056	BW25113; 842706_842710 delins AAACC	MAGE	
BC_S057	BW25113; 842706_842710 delins AGTAC	MAGE	
***trmD* mutants**			
BC_S089	BC_S049; *trmD* G117N	MAGE	All *trmD* mutant strains were stored transformed with pBAD-yTrm5 and pFREE. The plasmids were cured freshly before each experiment.<br><br>Y86* refers to 2,738,436_2,738,441 delins CACTAT, which gives TAA, TAG, and TGA stop codons in that order.
BC_S090	BC_S050; *trmD* G141A	MAGE	
BC_S092	BC_S052; *trmD* S165L	MAGE	
BC_S094	BC_S054; *trmD* D169A	MAGE	
BC_S095	BC_S055; *trmD* R154A	MAGE	
BC_S096	BC_S056; *trmD* Y115A	MAGE	
BC_S145	BC_S057; *trmD* Y86*	MAGE	
**Other BW25113 *trmD* wild-type derivatives**			
BC_S171	BW25113; *proS* E19K	MAGE	
BC_S189	BW25113; *proS* E19K	MAGE	
BC_S173	BW25113; *proS* G200S	MAGE	
BC_S184	BW25113; *proS* G200S	MAGE	
BC_S176	BW25113; *proS* E514V	MAGE	
BC_S177	BW25113; *proS* E514V	MAGE	
**BW25113 *trmD* G117N derivatives**			
BC_S148	S089; *trmD* c(-16)t	Isolated from population G117N-2-15	
BC_S150	S089; *trmD* c(-16)t	Isolated from population G117N-2-15	
BC_S147	S089; *trmD* c(-15)t	Isolated from population G117N-1-15	
BC_S152	S089; *trmD* g(-7)c	Isolated from population G117N-2-15	
BC_S155	S089; *trmD* g(-7)c	Isolated from population G117N-2-15	
BC_S149	S089; *trmD* c(-5)a	Isolated from population G117N-2-15	
BC_S151	S089; *trmD* L61V	Isolated from population G117N-2-15	
BC_S153	S089; *trmD* L61V	Isolated from population G117N-2-15	

**BW25113 *trmD* R154A derivatives**		
BC_S161	S095; *proS* E19K	Isolated from population R154A-2-22
BC_S164	S095; *proS* E19K	Isolated from population R154A-2-9
BC_S167	S095; *proS* E19K	Isolated from population R154A-2-9
BC_S159	S095; *proS* E19K, *argX* g(-5)a	Isolated from population R154A-2-22
BC_S160	S095; *proS* E19K, *argX* g(-5)a	Isolated from population R154A-2-22
BC_S162	S095; *proS* E19K, *argX* g(-5)a	Isolated from population R154A-2-22
BC_S165	S095; *proS* Y89N	Isolated from population R154A-2-9
BC_S166	S095; *proS* Y89N	Isolated from population R154A-2-9
BC_S168	S095; *proS* G107C	Isolated from population R154A-2-9
BC_S169	S095; *proS* G107C	Isolated from population R154A-2-9
BC_S156	S095; *proS* G200S	Isolated from population R154A-1-9
BC_S158	S095; *proS* G200S	Isolated from population R154A-1-9
BC_S157	S095; *proS* A231E	Isolated from population R154A-1-9
BC_S163	S095; *proS* A231E	Isolated from population R154A-1-9
BC_S170	S095; *proS* E514V	Isolated from population R154A-3-9
BC_S178	S095; *argV* t35g	MAGE
BC_S179	S095; *argV* t35g	MAGE
BC_S181	S095; *argX* g(-5)a	MAGE
BC_S209	S095; *argX* g(-5)a	MAGE

Supplementary Table 2. List of oligonucleotides used in this study.

[see attached file]

Supplementary Table 3. List of genomic mutations observed in evolved populations.

[see attached file]

Supplementary Table 4. Previously reported effects of TrmD substitutions on catalytic activity and bacterial growth.

Substitution	Effect on catalytic activity	Effect on growth	Notes
Y115A	8-fold decrease in k_{cat}/K_M for tRNA and 12-fold decrease in k_{cat}/K_M for SAM (Elkins et al., 2003).<br><br>6-fold increase in K_d for SAM, 34-fold increase in K_d for tRNA, and 22-fold decrease in k_{chem} (Christian et al., 2016).	Unknown.	-
G117N	Unknown; however, G117A abolishes activity (Elkins et al., 2003).	Reduces colony size to <10% of wild-type strain in *Salmonella enterica* Typhimurium (37 °C) (Björk et al., 2001).	G117S and G117Q *trmD* mutants of *S. enterica* Typhimurium are viable (Björk et al., 2001).
G141A	1400-fold decrease in k_{cat}/K_M for SAM (Elkins et al., 2003).<br><br>32-fold increase in K_d for SAM and 10-fold increase in K_d for tRNA with no effect on k_{chem} (Christian et al., 2016).	Unknown.	-
R154A	Abolishes activity (Elkins et al., 2003).<br><br>2-fold increase in K_d for SAM, 15-fold increase in K_d for tRNA, and 13-fold decrease in k_{chem} (Christian et al., 2016).<br><br>13,000-fold reduction in k_{cat}/K_M for tRNA in *Haemophilus influenzae* TrmD (Ito et al., 2015).	Unknown.	R154 forms two hydrogen bonds with guanine 37 of the tRNA substrate (Elkins et al., 2003).
S165L	Unknown; however, S165A substitution causes a 4-fold reduction in k_{cat}/K_M for tRNA	Reduces colony size to 10% of wild-type strain in	-

	in *H. influenzae* TrmD (Ito et al., 2015).	*S. enterica* Typhimurium (37 °C) (Björk et al., 2001).	
D169A	Abolishes activity (Elkins et al., 2003).<br><br>3-fold increase in K_d for SAM, 18-fold increase in K_d for tRNA, and 81-fold decrease in k_{chem} (Christian et al., 2016).<br><br>4100-fold reduction in k_{cat}/K_M for tRNA in *H. influenzae* TrmD (Ito et al., 2015).	Unknown.	D169 is the catalytic base responsible for deprotonation of N1 in guanine 37 of the tRNA substrate (Elkins et al., 2003).

Supplementary Table 5. Design of *trmD* mutations at the codon level. Mutagenic oligonucleotides were designed to introduce the indicated mutations into the *trmD* gene.

**Substitution**	**WT codon**	**Mutant codon**	**Amino acids accessible from mutant codon by single mutation**
Y115A	TAC	GCC	Val, Asp, Gly, Thr, Pro, Ser
G117N	GGT	AAT	Ile, Thr, Ser, Lys, Tyr, His, Asp
G141A	GGT	GCT	Val, Asp, Gly, Thr, Pro, Ser
R154A	CGG	GCG	Val, Glu, Gly, Thr, Pro, Ser
S165L	TCG	CTG	Met, Val, Pro, Gln, Arg
D169A	GAT	GCG	Val, Glu, Gly, Thr, Pro, Ser

Supplementary Table 6. List of genome rearrangements observed in evolved populations.

[see attached file]

Supplementary Table 7. Michaelis-Menten parameters for aminoacylation of $tRNA^{Pro}$ (CGG) by the WT and E514V variants of ProRS. Data represent mean ± s.d. of parameters estimated from three separate experiments (except for ProRS E514V with m^1G37 tRNA; two separate experiments). n.d., not determined.

tRNA	Enzyme	k_{cat} (s^{-1})	K_M (μM)	K_i (μM)	k_{cat}/K_M (M^{-1} s^{-1})
m^1G37	WT	0.40 ± 0.07	0.55 ± 0.01	2.41 ± 0.98	(7.31 ± 1.19) × 10^5
	E514V	0.84 ± 0.16	0.27 ± 0.09	0.85 ± 0.15	(3.22 ± 0.54) × 10^6
G37	WT	n.d.	n.d.	n.d.	(2.35 ± 0.68) × 10^4
	E514V	n.d.	n.d.	n.d.	(5.22 ± 1.17) × 10^5

References

Björk, G.R., Jacobsson, K., Nilsson, K., Johansson, M.J., Byström, A.S., and Persson, O.P. (2001). A primordial tRNA modification required for the evolution of life? *EMBO J. 20*, 231–239.

Christian, T., Sakaguchi, R., Perlinska, A.P., Lahoud, G., Ito, T., Taylor, E.A., Yokoyama, S., Sulkowska, J.I., and Hou, Y.-M. (2016). Methyl transfer by substrate signaling from a knotted protein fold. *Nat. Struct. Mol. Biol. 23*, 941–948.

Elkins, P.A., Watts, J.M., Zalacain, M., van Thiel, A., Vitazka, P.R., Redlak, M., Andraos-Selim, C., Rastinejad, F., and Holmes, W.M. (2003). Insights into catalysis by a knotted TrmD tRNA methyltransferase. *J. Mol. Biol. 333*, 931–949.

Ito, T., Masuda, I., Yoshida, K., Goto-Ito, S., Sekine, S., Suh, S.W., Hou, Y.-M., and Yokoyama, S. (2015). Structural basis for methyl-donor-dependent and sequence-specific binding to tRNA substrates by knotted methyltransferase TrmD. *Proc. Natl. Acad. Sci. 112*, E4197-205.

Kerpedjiev, P., Hammer, S., and Hofacker, I.L. (2015). Forna (force-directed RNA): Simple and effective online RNA secondary structure diagrams. *Bioinformatics 31*, 3377–3379.

Lorenz, R., Bernhart, S.H., Höner zu Siederdissen, C., Tafer, H., Flamm, C., Stadler, P.F., and Hofacker, I.L. (2011). ViennaRNA Package 2.0. *Algorithm Mol. Biol. 6*, 26.
